# Long non-coding RNA HOTTIP promotes renal cell carcinoma progression through the regulation of the miR-506 pathway

**DOI:** 10.18632/aging.205947

**Published:** 2024-06-19

**Authors:** Chengyao Zhang, Zhiya Hu, Yongxin Fu, Jiawu Wang

**Affiliations:** 1 Department of Thyroid Oncology, Chongqing University Cancer Hospital, Chongqing 400030, China; 2 Department of Urology, The Second Affiliated Hospital of Chongqing Medical University, Chongqing 400000, China

**Keywords:** HOTTIP, miR-506, renal cell carcinoma, ceRNA

## Abstract

HOXA transcript at the distal tip (HOTTIP), a lncRNA, induces cell proliferation and cancer progression. However, the expression and function of HOTTIP in renal cell carcinoma (RCC) were rarely reported. The role of the HOTTIP in RCC was explored in this study. HOTTIP expresses higher in RCC tissues than in normal tissues and indicates poor prognosis based on the TCGA database. The over- and low-expression HOTTIP cell line was established in this research to assess the oncogenic function of HOTTIP in RCC progression. Mechanistic analyses revealed that HOTTIP functioned as a competing endogenous RNA (ceRNA) for miR-506. RIP experiment and luciferase assay were performed to explore the mechanisms of the sponge between HOTTIP and miR-506. HOTTIP down-regulation attenuated cell proliferation, migration, and invasion, which could be rescued by miR-506 down-regulation. On the whole, this study revealed that the HOTTIP/miR-506 axis has a dominant impact on RCC progression and potentially provides a novel strategy for RCC diagnosis and therapy.

## INTRODUCTION

Renal cell carcinoma (RCC) is one of the most common tumors of the urinary system. The incidence and mortality of RCC are increasing year by year [1]. Although ultrasound and computed tomography have been widely used in clinical practice, about one-third of RCC patients have already developed local or distant metastases at first diagnosis, and these patients tend to have poor prognoses [2]. At the same time, RCC is not sensitive to traditional radiotherapy and chemotherapy, which brings great difficulties to clinical treatment [3]. Therefore, an in-depth understanding of the molecular mechanism of the occurrence and development of RCC and exploration of new potential therapeutic targets will help effectively control postoperative recurrence and metastasis and improve the survival level of patients.

As we all know, the research on miRNA has been increasing and deepening in the past 20 years, and the role and mechanism of miRNA in tumors are well known to us. MiRNAs are small non-coding RNAs of 20 to 25 nucleotides in length that are combined with messenger RNA (messenger RNA, mRNA) sequences (micro RNA response elements). Specific binding of MREs) inhibits the expression of its target genes [4]. Also, as an important member of the non-coding RNA family, how lncRNA exerts its biological functions in malignant tumors and how lncRNA interacts with miRNA to affect the malignant progression of tumors has become the focus of many scholars. In recent years, the ceRNA (competing for endogenous RNA) hypothesis has proposed that increase, as ceRNAs, bind competitively with miRNAs to affect gene silencing caused by miRNAs, regulate protein levels of coding genes, and participate in the expression regulation of target genes. This mode of ceRNA regulation has become a hot topic in the field of cancer research [5–9]. Salmena et al. [10] proposed the ceRNA hypothesis that mRNA, transcribed pseudogenes, and increase, as natural miRNA sponges, inhibit miRNA function by sharing one or more miRNA response elements in a complex and comprehensive regulatory network, thus affecting the occurrence and development of diseases. In this scientific context, Ballantyne et al. [11] found that lncRNA regulates miRNA through ceRNA sponge, and miRNA also binds to lncRNA and affects its stability.

Our research group successfully constructed ceRNA regulatory networks in all renal clear cell carcinoma and found that HOX family lncRNA-HOTTIP was a key gene in renal clear cell carcinoma, which was closely related to clinical prognosis [12]. Studies have shown that lncRNA located in cytoplasm can play a regulatory role as ceRNA [13, 14]. Combined with the ceRNA regulatory network we have constructed, it is speculated that lncRNA-HOTTIP may be transcribed and adsorbed miRNA regulatory target genes through a ceRNA “sponge”.

## RESULTS

### lncRNA-HOTTIP is over-expressed in RCC and associated with poor prognosis

**Figure 1:**
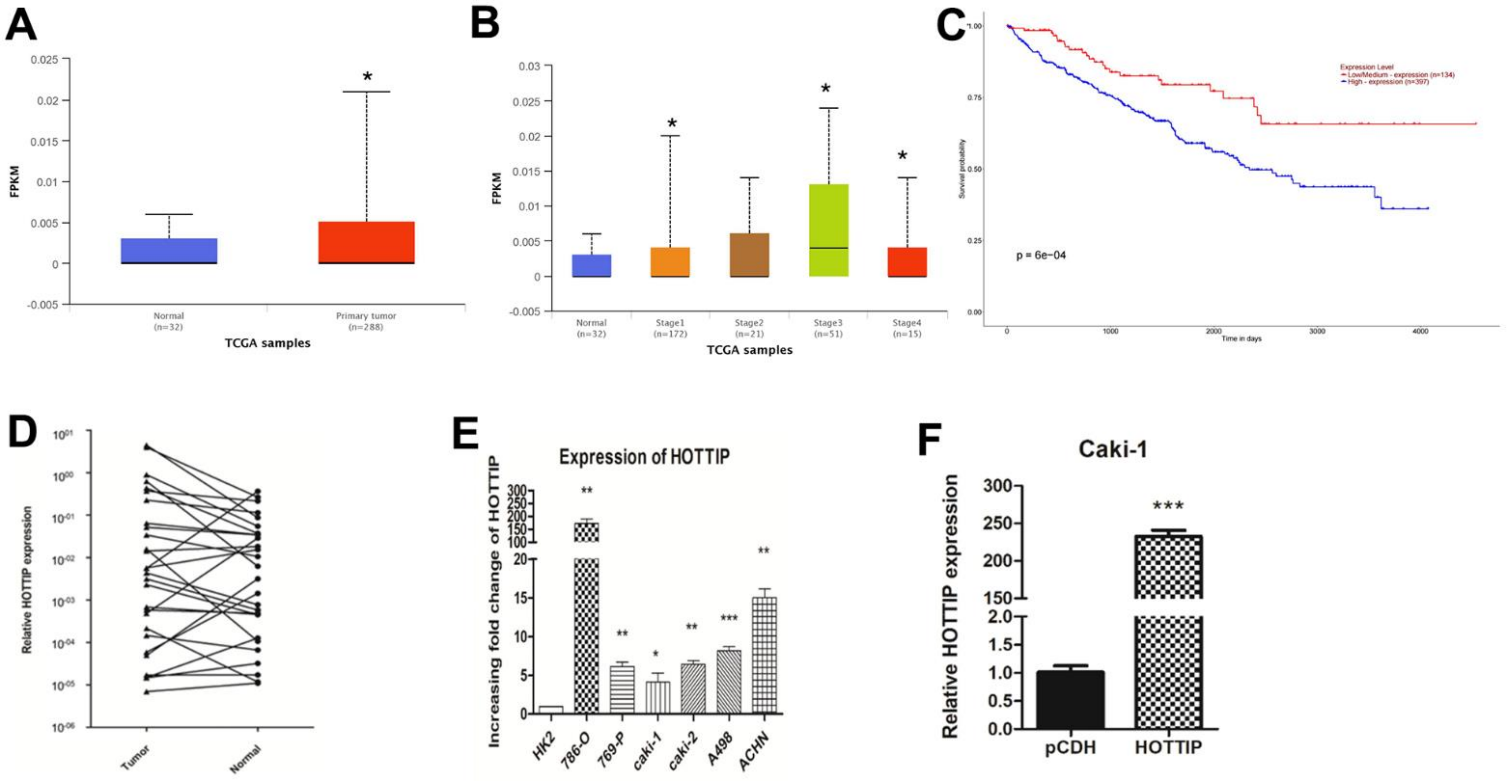
**lncRNA-HOTTIP is over-expressed in RCC and associated with a poor prognosis.** (**A**, **B**) TCGA database of tumor and paracancerous tissue HOTTIP expression; (**C**) Survival curves of patients with different HOTTIP expression; (**D**) RT-PCR results showed that lncRNA-HOTTIP expression was significantly up-regulated in RCC tissues; (**E**) The expression of lncRNA-HOTTIP was detected by RT-PCR, and the results showed that lncRNA-HOTTIP expression was significantly up-regulated in the RCC cell line, and in human normal renal tubular epithelial cells in the control group (*p < 0.05). (**F**) Expression efficiency of lncRNA-HOTTIP lentivirus transfected with Caki-1 was detected by RT-PCR.

According to the database from TCGA, we verified that lncRNA-HOTTIP was highly expressed in RCC tissues compared with normal tissue (Figure 1A, 1B). Next, we performed the overall survival (OS) analysis by Kaplan-Meier plotter. The chart showed that patients with high levels of lncRNA-HOTTIP had worse clinical outcomes (Figure 1C). The over-expression of lncRNA-HOTTIP was further confirmed using normal and tumor tissue from 29 patients (Figure 1D) and 6 RCC cell lines (Figure 1E). RCC cell lines (Caki-1) with stable overexpression of lncRNA-HOTTIP (HOTTIP) were constructed and screened for future experiments (Figure 1F).

### Over-expression of lncRNA-HOTTIP increases the proliferation, invasion, and migration of RCC

**Figure 2:**
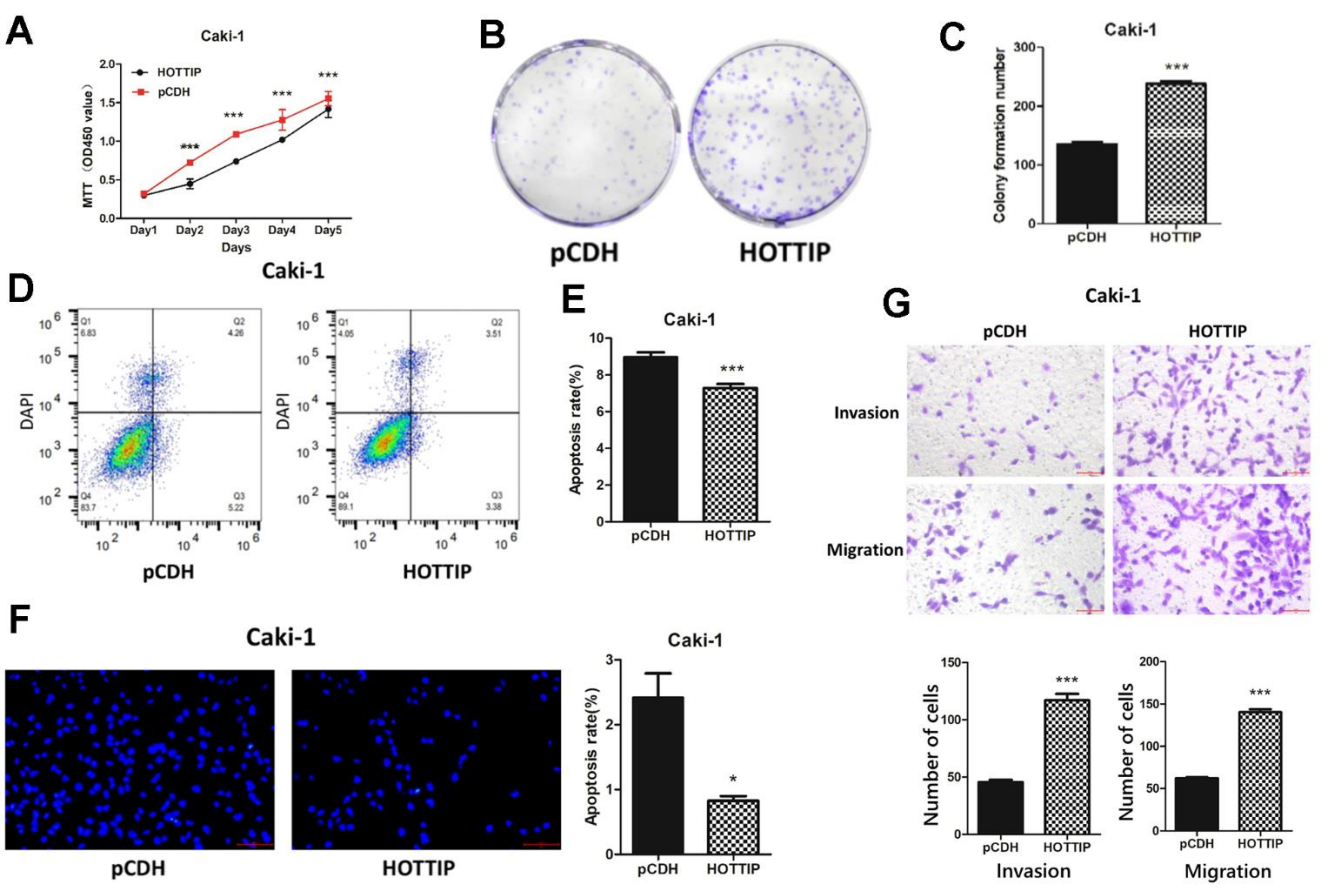
**Over-expression of lncRNA-HOTTIP increases the proliferation, invasion, and migration of RCC.** (**A**) The proliferation capacity of Caki-1-HOTTIP cells was enhanced by MTT compared with Caki-1-pCDH; (**B**, **C**) The proliferation capacity of Caki-1-HOTTIP cells was enhanced compared with that of Caki-1-pCDH cells by plate cloning assay; (**D**, **E**) The apoptosis of Caki-1-HOTTIP cells was decreased compared with Caki-1-HOTTIP cells by flow cytometry assay. (**F**) Hoechst apoptosis assay showed that the apoptosis of Caki-1-HOTTIP cells was weaker than that of Caki-1-pCDH cells. (**G**) Transwell test showed that the invasion and migration ability of Caki-1-HOTTIP cells were enhanced compared with Caki-1-pCDH cells.

The cells in Caki-1-HOTTIP absolutely increase in proliferation ability according to the MTT result at different time points (Figure 2A). The clone formation results reveal the increase in number and proliferation higher in comparison with the control group (Caki-1 cell with lentiviral-mediated normal control plasmid, Caki-1-pCDH) (Figure 2B, 2C). Moreover, the results of flow cytometry (Figure 2D, 2E) and Hoechst apoptosis assay (Figure 2F) further confirm a less apoptotic rate in Caki-1-HOTTIP cells. Transwell test showed that the invasion and migration ability of Caki-1-HOTTIP cells was enhanced compared with Caki-1-pCDH cells (Figure 2G). Therefore, we speculate that lncRNA-HOTTIP can promote the malignant progression of RCC, but its potential mechanism of action in RCC still needs to be further studied.

### Subcellular localization lncRNA-HOTTIP has the spatial conditions for ceRNA function

**Figure 3:**
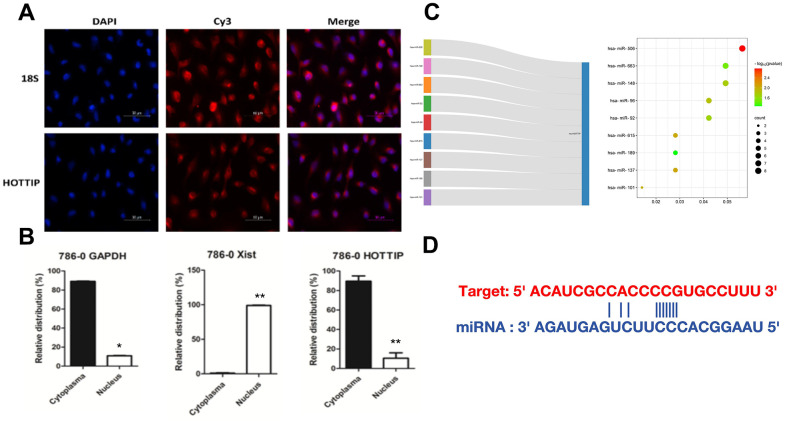
**Subcellular localization lncRNA-HOTTIP has the spatial conditions for ceRNA function.** (**A**) FISH detection found that lncRNA-HOTTIP was mainly located in RCC (786-O cytoplasm); (**B**) lncRNA-HOTTIP was mainly located in RCC (786-O cytoplasm); (**C**) The bubble chart predicted potential targeting miRNA of HOTTIP; (**D**) The potential combination site between miR-506 and lncRNA-HOTTIP.

The premise of the ceRNA regulatory model is based on lncRNA located in the cytoplasm. Our previous studies found that lncrNA-HOTTIP is a lncRNA located in the cytoplasm of RCC. Previous studies 786-0 cell line with high lncRNA-HOTTIP expression. In comparison with the negative control of 18s, the selection of Caki-1-HOTTIP by FISH (fluorescence *in situ* hybridization) technology proved that lncRNA-HOTTIP mainly existed in the cytoplasm in space (Figure 3A). The use of plasmolysis further confirmed that lncRNA-HOTTIP is mainly present in the cytoplasm (Figure 3B). As stated in the Introduction, increasing numbers of studies have suggested that lncRNAs may act as ceRNAs in regulating the biological functions of miRNAs. In this study, to further explore the underlying mechanisms of action of HOTTIP and its role in the development of RCC, we first examined a set of miRNAs that were predicted to bind HOTTIP using TargetScan. Bioinformatics analysis revealed that the HOTTIP transcript contained putative binding sites for miR-506 (Figure 3C, 3D).

### The oncogenic functions of HOTTIP are partially mediated through the negative regulation of miR-506

**Figure 4:**
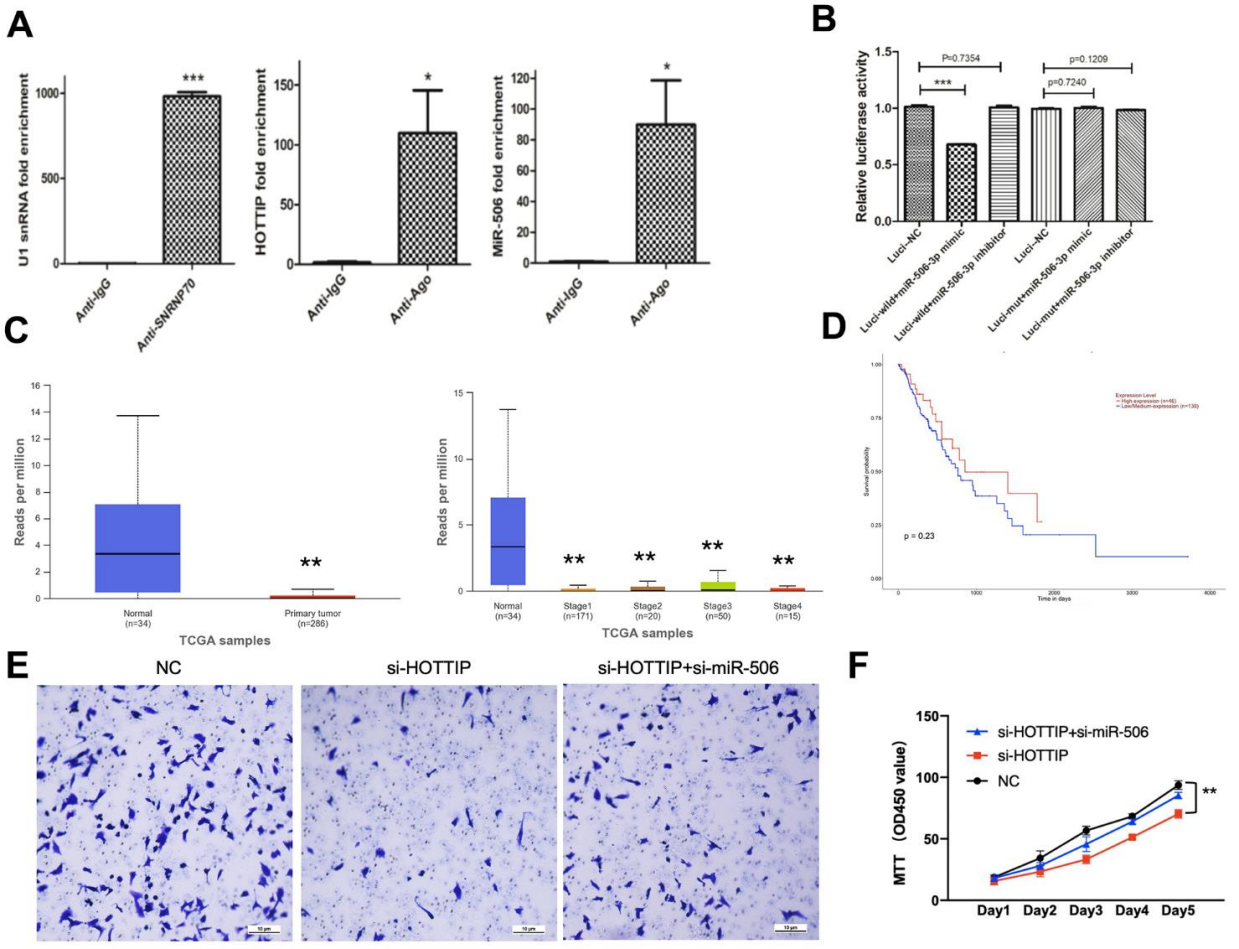
**The oncogenic functions of HOTTIP are partially mediated through the negative regulation of miR-506.** (**A**) In 786-O cells, RIP confirmed the interaction between lncRNA-HOTTIP and miRNA-506; (**B**) miRNA-506 can bind specifically to HOTTIP 3 ‘UTR; (**C**) TCGA database of tumor and paracancerous tissue miR-506 expression; (**D**) Survival curves of patients with different miR506 expression; (**E**) The invasion capacity of si-HOTTIP+si-miR-506 cells was enhanced by MTT compared with si-HOTTIP; (**F**) The proliferation capacity of si-HOTTIP+si-miR-506 cells was enhanced by MTT compared with si-HOTTIP.

Next, we verified the “sponge” adsorption between HOTTIP and miRNA-506 through RNA co-immunoprecipitation and double luciferase reporter gene technology (Figure 4A, 4B). According to the database from TCGA, we verified that miR-506 was low expressed in RCC tissues compared with normal tissue (Figure 4C). Next, we performed the overall survival (OS) analysis by Kaplan-Meier plotter. The chart showed that patients with low levels of miR-506 had worse clinical outcomes (Figure 4D). To verify the reciprocal repression of HOTTIP and miR-506, and to determine whether HOTTIP exerts its biological functions through miR-506, we performed a rescue experiment. We co-transfected si-HOTTIP and si-miR-506 into the 786-o cell line. The results revealed that the si-miR-506 significantly reversed the suppressive effects exerted by the knockdown of HOTTIP on cell proliferation and invasion of Caki-1 cells (Figure 4E, 4F), suggesting that HOTTIP plays its oncogenic role in RCC cells through miR-506. Furthermore, the potential target protein and relative combination site were predicted using TargetScan, and the combination site was confirmed using dual-luciferase. The results showed that direct action and key binding sites of miRNA-506 and its downstream target gene VIM were detected, and TGCCTTA was deleted by metamorphic granules (Supplementary Figure 1A, 1B). The results showed that miRNA-506 had a direct effect on VIM and a key binding site.

## DISCUSSION

The function of lncRNAs, such as carcinogenesis, has been widely reported in many biological functions. As for RCC, former research has reported that many lncRNAs play key roles in tumorigenesis. Yihui et al. revealed that IGFL2-AS1 enhanced sunitinib resistance in RCC by increasing autophagy [15]. Guo et al. revealed that suppression of HOTAIR alleviates RCC through the miR-126 pathway [16]. In our research, the functional role and clinical potential of HOTTIP in RCC were explored. Despite the former research of HOTTIP, little is known about the molecular mechanism. HOTTIP, a widely reported oncogene, expresses highly in RCC tissues compared with normal tissues. The high expression of HOTTIP also relates to poor clinical outcomes. *In vitro*, results confirmed that HOTTIP could simultaneously increase proliferation, migration, and invasion in RCC. Furthermore, the mechanism of HOTTIP was explored.

The ceRNA theory provides the most theory regarding lncRNA, which functions as a sponge to regulate miRNA expression. The former study has reported that HOTTIP coordinates with miR-196 to underpin leukemogenesis through FAS signaling [17]. Wang et al. reported that HOTTIP induces acute myocardial infarction by regulating miR-92a [18]. A competing combination was also revealed between HOTTIP and miR-192 [19]. In this study, we discovered that HOTTIP and miR-506 negatively regulated each other and were involved in the ceRNA regulatory network. The reasons for choosing miR-506 are shown below: Firstly, the expression of miR-506 in normal and tumor tissue are significantly different and the expression of miR506 positively correlates with the survival curve. Secondly, also in Figure 3C, we predict the potential target of HOTTIP. According to the bubble chart, miR-506 is the predicted potential targeting miRNA of HOTTIP with the highest influence and combination rate. HOTTIP expression was suppressed by miR-506 mimics. Due to HOTTIP functioning as an endogenous miRNA sponge to bind to miR-506; the inhibition of HOTTIP abrogated this sponge, leading to an increase in miR-506 expression.

Former research regarded miR-506 as a tumor suppressive role in various tumors [20–22]. However, its role in RCC has not yet been investigated, at least to the best of our knowledge. In this study, we revealed that miR-506 targets both mRNA and lncRNA HOTTIP. Accordingly, the identification of HOTTIP and miR-506 as each other’s targets expands the repertoire of miR-506 targets. Meanwhile, the expression of miR-506 could be suppressed by HOTTIP via the combination in the 3-UTR site, which forms a reciprocal negative regulatory loop. These results provide further supporting evidence of the ceRNA regulatory network. As for biological function in RCC, our result revealed that suppression of miR-506 could partially rescue cell proliferation, invasion, and migration, which are attenuated by HOTTIP down-regulation. In summary, these data strongly suggest that HOTTIP affects the biological function in RCC cells by directly targeting, and negatively regulating miR-506.

Taken together, we revealed that HOTTIP, as a miRNA sponge, could suppress the function of miR-506 and consequently induce carcinogenesis and function in RCC. Due to this crucial role that HOTTIP plays in the progression of RCC, it may thus serve as a therapeutic target, as well as a prognostic biomarker for RCC.

## MATERIALS AND METHODS

TCGA analysis of HOTTIP expression in tumors and samples from RCC patients and construction of survival curves. mRNA or miRNA expression data were obtained from the TCGA database (https://www.cancer.gov/ccg/research/genome-sequencing/tcga). The SVA package was used for batch correction. In order to identify differentially expressed mRNAs (DEmiRNAs), the edgeR package (criteria: |logFC| > 2.5, padj < 0.05) was used to compare differences between the control group and the tumor group. Additionally, mRNA profiles (normal samples: 32, tumor samples: 288) were obtained from the UCSC clinical database (http://genome.ucsc.edu/). The volcano plot was drawn by the R package ‘cluster profiler’. The edgeR package (criteria: |logFC| > 1, padj < 0.05) was used to analyze DEmRNAs between the normal group and tumor group. We get the analysis result of survival comparison by using the Kaplan-Meier plotter method through the TCGA data (GraphPad Prism 8).

The ceRNA network was established according to our former research [12]. Briefly described, a coexpression network of DEmRNAs, lncRNAs, and miRNAs was constructed and visualized using the Cytoscape v 3.5.1 software. Interactions between DElncRNA and miRNA pairs were predicted based on miRcode (http://www.mircode.org/). The regulatory relationships between DEmiRNA and DEmRNA pairs were retrieved from miRTarBase (https://mirtarbase.cuhk.edu.cn/~miRTarBase/miRTarBase_2022/php/index.php), miRDB (http://www.mirdb.org/), and TargetScan (http://www.targetscan.org/vert_71/). The regulatory relationships between miRNAs and their target mRNAs were verified by experimental methods, including reporter assays, quantitative real-time polymerase chain reaction, western blot analysis, microarrays, and next-generation sequencing in miRTarBase.

### Construct a stable cell line with lncRNA-HOTTIP overexpression

CAKi-1 cells were transfected with lentivirus, and CAKi-1-HOTTIP and KAKi-1-HOTTIP-CTRL cells that stably overexpressed lncRNA-HOTTIP were selected by puromycin. The overexpression efficiency of lncRNA-HOTTIP in Caki-1-Hottip and Caki-1-HOTTIP CTRL was verified by qRT-PCR at the RNA level.

### Changes in cell biological behavior of overexpressed stable cell lines were detected

The proliferation, invasion and metastasis of lncRNA-HOTTIP cells in AKIi-1-Hottip and AKIi-1-Hottip-CTRL cells were detected by MTT, plate clone formation assay, flow cytometry assay, Hoechst apoptosis assay, Transwell migration and invasion assay, and tumor transplantation in nude mice The effect of lncRNA-HOTTIP on the proliferation and metastasis of RCC cells was verified.

### The mechanism of lncRNA-HOTTIP/miRNA-506 ceRNA regulatory network in RCC

Localization of lncRNA-HOTTIP in RCC cells by FISH. The fluorescein-labeled probe was used to enter 786-O cells. Based on complementary pairing and annealing to form a double strand, the lncRNA-HOTTIP that could bind to the probe was labeled with fluorescence and its location was displayed. The location and relative expression of lncRNA-HOTTIP were detected by nucleic acid complementary pairing characteristics.

A nucleoplasmic separation assay was performed using the PARISTM Kit, 2X lysis/binding solution was heated at 37° C until completely dissolved, an equal volume of lysis solution was mixed, 786-O cell lysate was eluted at 95° C, RNA isolation and protein analysis were performed. The main localization of lncRNA-HOTTIP was further examined.

### To verify the mechanism of lncRNA-HOTTIP “sponge” adsorbing miRNA-506

1. The direct binding sites of lncRNA-HOTTIP and miRNA-506 were verified by dual luciferase reporter gene: Dual luciferase reporter plasmids containing the full-length sequence of HOTTIP (Luci-wild) and the mutant sequence of miR-506-3p binding site on HOTTIP (Luci-mut) were constructed. The luciferase activity was monitored to determine the expression change of the reporter gene and quantitatively reflect the inhibitory effect of lncRNA-HOTTIP on miRNA-506-3p, that is, whether lncRNA-HOTTIP binds to miRNA-506-3p or not.
2. The direct binding site between miRNA-506 and target gene VIM was verified by dual luciferase reporter gene: A dual luciferase reporter gene vector containing the full-length sequence of VIM (Luci-wild) and the mutant sequence of miR-506-3p binding site on VIM (Luci-mut) was constructed. By comparing the overexpression or interference of miRNA-506, the expression of the reporter gene was determined by monitoring the luciferase activity. The inhibitory effect of miRNA-506 on VIM was quantified, and the fluorescence value was used to determine whether miRNA-506 binds to the target gene of VIM.
3. The effect of lncRNA-HOTTIP “sponge” on miRNA-506 adsorption was further verified by RIP: Millipore Magna RIP™ kit was used for the RIP experiment. The lncRNA-miRNA complex in the cells was precipitated by the specific binding of the antibody to the target RNA, and the RNA in the lncRNA-miRNA complex was detected.

### The effects of lncRNA-HOTTIP and miRNA-506 on the proliferation, invasion, and metastasis of RCC cells were verified by co-transfection

1. Caki-1 cells were transfected with lentivirus and stably overexpressed lncRNA-HOTTIP was obtained by puromycin selection. In Caki-1 cell line stably overexpressing lncRNA-HOTTIP, miRNA-506 mimic was co-transfected as follows: These are called HOTTIP+mir-506 mimic, HOTTIP-Ctrl+mir-506 mimic, HOTTIP+Ctrl mimic, HOTTIp-Ctrl +Ctrl mimic.
2. HOTTIP+mir-506 mimic and HOTTIP-Ctrl+mir-506 mimic were detected by MTT, Transwell migration and invasion assay, and other experimental methods. HOTTIP+Ctrl mimic, HOTTIP-Ctrl+Ctrl mimic changes in cell proliferation invasion and metastasis.

### Availability of data and materials

The datasets used and/or analyzed during the current study are available from the corresponding author on request.

## Supplementary Material

Supplementary Figure 1
