# Supplementary material for: Long non-coding RNA HOTTIP promotes renal cell carcinoma progression through the regulation of the miR-506 pathway
Source: Aging (Albany NY). 2024 Jun 19;16(13):10832–40. doi: 10.18632/aging.205947 (PMC11272121; doi:10.18632/aging.205947)
Supplement: Supplementary Figure 1 [file aging-16-205947-s001.pdf]

SUPPLEMENTARY FIGURE

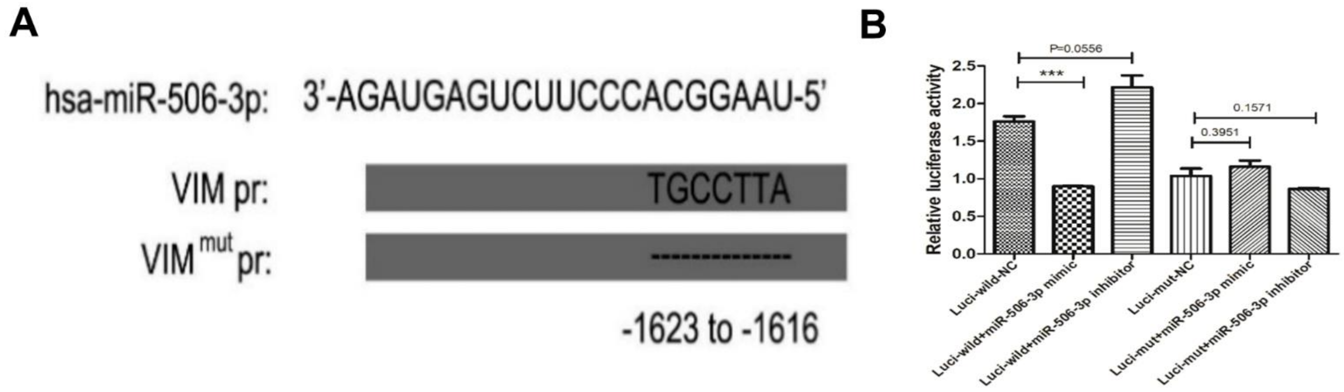

Supplementary Figure 1. (**A**) There is a binding site for mir-506 at 1623 upstream of the transcription start site (TSS) in VIM. (**B**) Dual-luciferase reporter gene assay was used to detect the change in luciferase activity in the mir-506 mimics and mir-506 inhibitors groups.
